# Brain Structural Abnormalities in Patients with Post-COVID-19 Headache

**DOI:** 10.3390/neurolint17040050

**Published:** 2025-03-26

**Authors:** Klaudia Széphelyi, Szilvia Kóra, Gergely Orsi, József Tollár

**Affiliations:** 1 Doctoral School of Health Sciences, Faculty of Health Sciences, University of Pécs, 7622 Pécs, Hungary; silviasabokora@gmail.com (S.K.); tollarjozsef86@gmail.com (J.T.); 2 Faculty of Health Sciences and Social Studies, Department of Theoretical Health Sciences and Health Management, University of Szeged, 6726 Szeged, Hungary; 3 Department of Neurology, Medical School, University of Pécs, 7622 Pécs, Hungary; gergo.orsi@gmail.com; 4 HUN-REN-PTE Clinical Neuroscience MR Research Group, Hungarian Research Network, 7622 Pécs, Hungary; 5 Somogy County Móricz Kaposi Teaching Hospital, 7400 Kaposvár, Hungary; 6 Digital Development Center, Széchenyi István University, 9026 Győr, Hungary; 7 Department of Otorhinolaryngology-Head and Neck Surgery, University of Pécs Medical School, 7622 Pécs, Hungary; 8 János Bolyai Research Scholarship of the Hungarian Academy of Science, 1051 Budapest, Hungary; 9 Faculty of Health Sciences, University of Pécs, 7622 Pécs, Hungary

**Keywords:** COVID-19, headache, MRI, brain, white matter lesion

## Abstract

Background/Objectives: Headache is one of the most common neurological symptoms associated with COVID-19, affecting approximately 25% of patients. While most headaches resolve within weeks, some persist for months, suggesting underlying structural brain changes. This study aimed to identify brain MRI abnormalities associated with chronic headaches in patients with a history of COVID-19 infection. Methods: This retrospective study included 30 patients with post-COVID-19 headaches and 30 control patients with no history of COVID-19. Demographic characteristics were analyzed using *t*-tests and chi-square tests. MRI findings were categorized into six types: cortical atrophy, white matter lesions, vascular lesions, lacunar lesions, vascular encephalopathy, and sinusitis. Differences in MRI findings between the two groups were evaluated using chi-square tests. Secondary outcomes included the analysis of symptoms accompanying headaches, diagnoses following MRI, and treatments applied. Results: White matter lesions were significantly more frequent in the post-COVID-19 group (50%) compared to controls (20%) (*p* = 0.015). Conversely, sinusitis was more prevalent in the control group (36.7%) than in the post-COVID-19 group (6.7%) (*p* = 0.005). Other MRI abnormalities showed no significant differences. Cognitive dysfunction (30%) and dizziness (33.3%) were the most common associated symptoms. The most frequent diagnoses after MRI in the post-COVID-19 group were headaches/migraines (23.3%), post-COVID-19 headache (20%), and vestibular syndrome (13.3%). Conclusions: Persistent post-COVID-19 headaches may be linked to structural white matter changes observed in MRI. Further research, ideally including pre-infection imaging data, is needed to determine the causal relationship between these lesions and chronic headache symptoms. Trial Registration: This study was registered in ClinicalTrials with the trial registration number NCT06825741 on 13 February 2025.

## 1. Introduction

The clinical manifestations of the Coronavirus Disease 2019 (COVID-19) pandemic are heterogeneous and may include respiratory symptoms (dyspnea, chest pain, and cough), gastrointestinal symptoms (diarrhea and vomiting), musculoskeletal symptoms (myalgia and arthralgia), and neurological symptoms (headache, vertigo, anosmia, and ageusia) [1,2]. Approximately 25% of patients report headache as a neurological symptom, making it the fifth most common symptom [3]. The characteristics of post-COVID-19 headaches most closely resemble tension-type headaches and migraines [4]. It occurs in the upper/frontal part of the head; it is usually persistent and does not respond to treatment [5]. The average duration of headache is around two weeks; however, in many cases, it can become chronic and is still present after 2 months [6]. Given the high prevalence of migraine in the general population, it is anticipated that headaches triggered by COVID-19 will be more common in individuals with a history of headache disorders. It is often difficult to recognize whether the headache experienced by patients is really an exacerbation of their known headache disorder, a new secondary headache, or a new headache syndrome due to an infection. Nonetheless, as typical headaches in patients with primary headache disorders can be exacerbated by systemic illnesses, such as viral infections, it is imperative to thoroughly evaluate patients with previously diagnosed headache conditions [7].

Most brain-imaging studies related to COVID-19 have primarily focused on acute cases, individual radiological reports, or case series. Imaging techniques such as computer tomography (CT), positron emission tomography (PET), and magnetic resonance imaging (MRI) have widely revealed severe brain abnormalities, including hyperintensity in white matter, cerebral hypoperfusion, and signs of ischemic events [8,9]. Among the larger studies addressing cerebrovascular damage, most have not identified clear pathological markers in the majority of patients, nor have they found a consistent spatial pattern for the distribution of white matter hyperintensities or microbleeds [10,11]. MRI is one of the most suitable methods for assessing structural changes in the brain behind a headache because soft tissues can be examined noninvasively and without radiation exposure. This includes clear visualization of gray and white matter, brain ventricles, blood vessels, and nerves [12]. The standard protocol typically includes the following sequences: T2-weighted, T2-weighted Fluid-Attenuated Inversion Recovery (FLAIR), Diffusion Weighted Imaging (DWI) axial, T1-weighted coronal, and T2-weighted sagittal planes. If necessary, post-contrast 3D sagittal T1-weighted imaging is performed. In cases of headache, time-of-flight (TOF) imaging is often added to the protocol [13].

The aim of our research is to reveal the structural abnormalities behind the headache in patients with the disease COVID-19, which can be diagnosed during cranial MRI.

## 2. Materials and Methods

### 2.1. Participants and Design

In this retrospective study, patients were enrolled from the outpatient clinic of Szent Margit Hospital between September 2020 and December 2022.

In the control group, the patients did not have a history of COVID-19 infection in their medical records. Exclusion criteria included the presence of any neurological disease or complaint in the patient’s medical history. A total of 30 control participants were enrolled. The mean age of the participants was 61.07 ± 17.48 years (age range 27–91 years). In the control group, we only examined the MRI findings, without considering the medical history or the post-MRI results.

In the post-COVID-19 headache group, the inclusion criteria were adult age (>18 years) and a previous history of COVID-19 infection, along with a requested brain MRI examination by the attending physician due to headaches. Exclusion criteria included the presence of any neurological disease or complaint in the patient’s medical history. A total of 30 participants were enrolled. The mean age of the participants was 55.03 ± 15.51 years (age range 27–79 years). We reviewed the patients’ medical histories and the time elapsed between COVID-19 infection and MRI. We also examined the presence of other relevant symptoms accompanying the headaches. The presence of the following MRI abnormalities was also recorded: cortical atrophy, white matter lesions, vascular lesions, lacunar lesions, vascular encephalopathy, and sinusitis. The final diagnoses and administered treatments following the MRI were also recorded.

### 2.2. MRI Protocol

The MRI parameters are summarized in Table 1. The brain MRI protocol consisted of the following measurements:

- Sagittal T1-weighted;
- Coronal FLAIR;
- Axial T2-weighted;
- Axial DWI;
- Axial Susceptibility Weighted Imaging (SWI);
- Axial TOF;
- Optional: contrast enhanced 3D sagittal T1-weighted.

### 2.3. Outcomes

**Figure 1:**
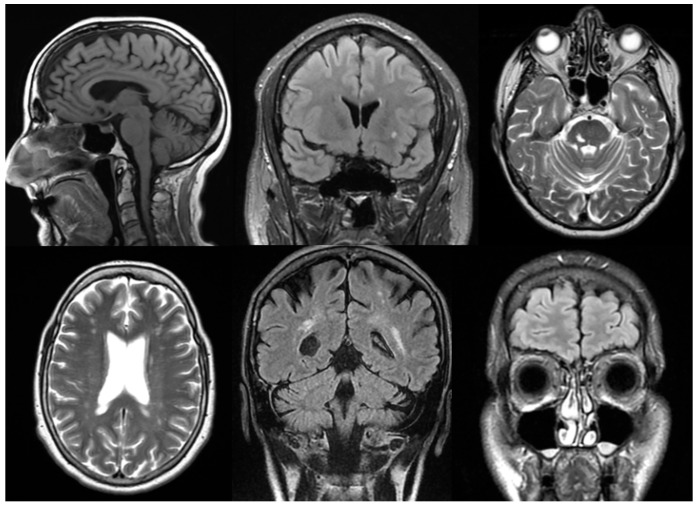
From left to right in the first row are cortical atrophy, white matter lesion, and vascular lesions and, in the second row, are lacunar lesion, vascular encephalopathy, and sinusitis.

The primary outcomes are the structural changes observed on MR images. Throughout the study, all findings were systematically evaluated by a neuroradiologist. To diagnose various pathologies, neuroradiologists in Hungary apply local professional guidelines and the recommendations of the National Health Professional College. The most common pathologies were diagnosed based on the following criteria (Figure 1).

- Cortical atrophy: the Scheltens scale aids in the standardized grading of atrophy severity. It is characterized by a reduction in cortical thickness, widening of the sulci, and dilation of the ventricular system, particularly the lateral ventricles. These changes are typically observed on T1-weighted and FLAIR images, where thinning of the cortical structures is evident [14].
- White matter lesions: the Fazekas scale and the Standards for Reporting Vascular Changes on Neuroimaging (STRIVE) criteria are the most commonly used standards. White matter lesions are identified on FLAIR and T2-weighted images as bright (hyperintense) areas located in the periventricular or deep white matter. These may appear as discrete spots or confluent regions, typically indicating small vessel disease [15].
- Vascular lesions: in Hungary, the European Stroke Organization Guidelines serve as the primary reference. MR angiography is routinely used to assist in the diagnosis of vascular abnormalities. On DWI, acute ischemic lesions appear as hyperintense signals. T1/T2-weighted and magnetic resonance angiography (MRA) images can identify stenosis, occlusions, or vascular wall abnormalities. In cases of hemorrhage, hemosiderin deposits can be detected using SWI techniques [16].
- Lacunar lesions: the STRIVE criteria assist in identifying these lesions. They are characterized by small (<15 mm) round or oval hyperintense areas on T2/FLAIR images. Common locations include the thalamus, basal ganglia, and internal capsule. On T1-weighted images, they appear as hypointense signals [15].
- Vascular encephalopathy: the Hungarian Stroke Guidelines and the recommendations of the MSKT (Hungarian Stroke Consensus Council), along with the application of the Fazekas scale, are essential for evaluation. Diffuse white matter hyperintensities are observed on FLAIR images. Subcortical infarcts, dilated perivascular spaces, or signs of microvascular disease may also be present. Cortical and subcortical atrophy is frequently associated with ventricular enlargement [17].
- Sinusitis: the American College of Radiology (ACR) Appropriateness Criteria and the Lund-Mackay scoring system are utilized. Sinus opacification, mucosal thickening, or fluid levels are identified on T2-weighted images. In acute inflammation, high-intensity signals and fluid levels are observed on T2. In chronic sinusitis, findings include thickened bony walls, fibrosis, or the presence of mucoceles [18].

Secondary outcomes are other symptoms appearing in addition to headache, the final diagnosis after MR imaging, the treatments applied after the MR examination, and the time elapsed between COVID-19 infection and the MRI.

### 2.4. Statistical Analyses

Demographic characteristics of the groups were compared to prove that age and gender distribution were similar. Age was analyzed using an independent samples *t*-test, while gender distribution was assessed with a chi-square test. Normality was assessed using the Shapiro–Wilk test, and the data followed a normal distribution.

Descriptive statistics were used to summarize the baseline characteristics. Data are reported as mean ± SD.

To evaluate differences in MRI findings between the control and post-COVID-19 headache groups, chi-square tests were conducted for cortical atrophy, white matter lesions, vascular lesions, lacunar infarcts, vascular encephalopathy, and sinusitis/sinus abnormalities.

Since MRI-related diagnoses and therapies were not assessed in the control group, we focused on secondary outcomes, analyzing the frequencies of secondary findings to provide insights into their distribution and potential relevance.

The significance level was set at *p* < 0.05 for all analyses.

Statistical calculations were conducted using the IBM Corp. released 2019, IBM SPSS Statistics for Windows, Version 26.0. Armonk, NY, USA: IBM Corp.

### 2.5. Ethical Considerations

This study was registered at ClinicalTrials.gov under the identifier NCT06825741 on 13 February 2025. The study was approved by the Regional Ethical Committee of Szent Margit Hospital (IG/296-1/2025/1). The study was conducted according to the World Medical Association Declaration of Helsinki. As it was a retrospective study, informed consent was waived in accordance with institutional guidelines. Data collection complied with General Data Protection Regulation (GDPR) and patient confidentiality regulations.

## 3. Results

### 3.1. Baseline Characteristics

Table 2 shows the descriptive data in the post-COVID-19 headache group. This study involved 10 male and 20 female participants, with an average age of 55.03 ± 15.51 years. The participants’ ages ranged from 27 to 79 years. Time between COVID-19 infection and MRI, other post-COVID-19 symptoms and, MRI results are discussed in detail in the outcome results.

Table 3 shows the descriptive data in the control group. This study involved 15 male and 15 female participants, with an average age of 61.07 years (±17.48). The participants’ ages ranged from 27 to 91 years. MRI results are discussed in detail in the outcome results.

No significant differences were found between the control and post-COVID-19 headache groups for age (t (58) = 1.414, *p* = 0.163) or gender distribution (χ^2^ (1, N = 60) = 1.714, *p* = 0.190).

### 3.2. Primary Outcome

In total, 85% of the enrolled subjects had positive MRI findings (structural changes). The MR results are summarized in Table 4.

In the post-COVID-19 headache group, white matter lesions (50%) were the most commonly observed structural change, followed by cortical atrophy (23.3%) and vascular encephalopathy (23.3%).

Significant differences were detected in white matter lesions (*p* = 0.015) and sinusitis/sinus abnormalities (*p* = 0.005). Other MRI findings, including cortical atrophy, vascular lesion, lacunar lesion, and vascular encephalopathy, did not show statistically significant differences (*p* > 0.05).

### 3.3. Secondary Outcome

In addition to headaches, the two most prevalent symptoms were dizziness (33.3%) and cognitive disorders (30%). Other reported symptoms included sleep disorders, balance disturbances, loss of consciousness, fatigue, tinnitus, nausea, and palpitations, but these symptoms occurred in only a few patients.

The diagnoses after MRI are summarized in Table 5.

In six patients, no diagnosis was provided. Among the remaining 24 cases, the most frequently diagnosed conditions were headache, migraine (23.3%), post-COVID-19 headache (20%), and vestibular syndrome (13.3%). Unfortunately, we do not have detailed information on the specific criteria used by the treating physicians to determine the final diagnoses. Given that post-COVID headache is a relatively new clinical entity, standardized diagnostic criteria may not yet be fully established or consistently applied in clinical practice. This could explain why some patients who may have had post-COVID headache were instead diagnosed with other headache disorders, such as migraine.

The association between MRI findings and diagnoses in the post-COVID-19 headache group are summarized in Table 6.

The data show that white matter lesions and cortical atrophy occur in most diagnoses. For example, white matter lesions were observed in 50% of patients in the post-COVID-19 group, but they were also present in 57.1% of the migraine and other headache group.

Interestingly, the patient diagnosed with visual impairment exhibited only cortical atrophy, while the patient with dementia showed all observed structural changes.

The applied treatments are summarized in Table 7.

Cognitive-function-enhancing drugs were the most frequently prescribed (23.3%), followed by antidepressants (20%) and anti-inflammatory medications (10%). Additionally, a notable proportion of patients (10%) received lifestyle modification advice.

## 4. Discussion

This study investigates the underlying structural brain changes associated with chronic headaches following COVID-19.

One of our most noteworthy findings is that white matter lesions were identified in 50% of the post-COVID-19 group, compared to 20% in the control group. Given that patients in the post-COVID-19 group had no neurological symptoms prior to the infection, it is likely that these lesions were a consequence of COVID-19. Previous studies have also shown that COVID-19 can cause white matter changes during the acute phase; however, few studies have explored the long-term resolution of these changes. Since brain lesions can increase the risk of cognitive decline, white matter lesions may contribute not only to headaches but also to cognitive disorders [5,7,8,19,20]. Future research should consider examining the precise locations and numbers of these white matter lesions.

Although our results did not show a significant difference, cortical atrophy was the second most common finding in the post-COVID-19 group in our study. This aligns with other studies that have reported cortical atrophy as a potential factor contributing to post-COVID-19 headaches and associated cognitive disorders as concomitant symptoms [7].

While headaches are present as a symptom during COVID-19 infection, they become chronic in a significant percentage of cases and can persist after the infection subsides. Consistent with our findings, other studies have noted that patients seek medical attention for headaches approximately 3–4 months after COVID-19 infection [5,11].

Likewise, dizziness, cognitive dysfunction, and fatigue have been identified as accompanying symptoms in other studies [5,9,10].

The psychological burden associated with the COVID-19 pandemic should not be overlooked, as it may also contribute to headaches. Given that some studies have demonstrated an association between post-COVID-19 headaches and psychological issues, it is essential for healthcare providers to offer not only pharmacological treatments for headache but also lifestyle advice and antidepressants to facilitate a quicker recovery. However, some research has indicated that the use of anti-inflammatory drugs does not improve symptoms [3,9].

Unfortunately, during data collection, we were unable to obtain information regarding the phenotype of the headaches, as this was not included in the outpatient referral forms. It is crucial for healthcare providers to place greater emphasis on the characteristics of headaches (e.g., migraine or tension-type), as this can serve as a valuable starting point for treatment selection [11].

Given that the 30 examinations incurred a cost of HUF 2.462.439 (Hungarian Forint) on the healthcare system, it is vital to demonstrate the actual role of MRI in the diagnosis and management of post-COVID-19 headaches. If white matter lesions caused by COVID-19 are indeed responsible for headaches, it is important to clarify whether the location of these lesions affects treatment outcomes. If the information obtained from MRI (such as localization and number of lesions) does not influence treatment decisions, then eliminating MRI could reduce healthcare expenses while also shortening wait times.

## 5. Limitation

One of the main limitations of this study is the relatively small sample size, which may impact the generalizability and statistical power of our findings. However, our study provides a solid foundation for future research, allowing for further investigations with larger cohorts to validate and expand upon our results. Additionally, we did not investigate whether the headaches were present prior to the COVID-19 infection through direct historical documentation. However, none of the included patients had undergone medical evaluation for headache before their COVID-19 infection, nor was there any record of prior headache-related complaints in their medical history. Therefore, we assumed that the headaches observed in our study emerged after the infection. We also did not examine the localization of the lesions identified during the MRI.

## 6. Conclusions

A significant proportion of patients who have recovered from COVID-19 continue to experience headaches 3–4 months post-infection. The presence of white matter lesions in these patients suggests a potential link between post-COVID-19 headaches and structural brain changes. Given the importance of this issue, further research is warranted. The significance of the findings could be further enhanced by including patients who had prior brain MRI scans before their COVID-19 infection, which would allow for a more precise assessment of changes attributable to the infection.

## Figures and Tables

**Table 1:** MRI sequences.

	Sequence Type	TR ^1^	TE ^2^	TI ^3^	FOV ^4^	Matrix ^5^	FS ^6^	Scan Time ^7^	NEX ^8^	b-Value ^9^	Bandwith ^10^
**Sagittal T1**	FSE ^11^	577	12.34	-	240	512 × 512	N	3.5	1	-	31.25
**Axial T2**	FSE	7580	86.5	-	260	512 × 512	N	3.5	1.5	-	62.5
**Coronal T2**	FLAIR	9000	120	2580	240	256 × 256	N	3.5	1	-	70
**Axial DWI**	SE EPI ^12^	7551	83.1	-	260	256 × 256	N	2.5	1	20/1000	250
**Axial SWI**	GRE ^13^	73.7	47.07	-	260	512 × 512	N	3	1	-	41.67
**Axial TOF**	SPGR ^14^	26	6.8	-	220	512 × 512	Y	3.5	1	-	250
**+c Sagittal 3D T1**	FSPGR ^15^	6.884	2.06	-	240	512 × 512	N	3.5	1	-	83.33

^1^ TR—Repetition time (ms); ^2^ TE—Echo Time (ms); ^3^ TI—Inversion Time (ms); ^4^ FOV—Field of View (mm); ^5^ Matrix (pixel); ^6^ FS—Fat Saturation (N—no, Y—yes); ^7^ Scan Time (min); ^8^ NEX—Number of Excitations; ^9^ b-value (s/mm^2^); ^10^ Bandwidth (kHz); ^11^ FSE—Fast Spin Echo; ^12^ SE EPI—Spin Echo Echo Planar Imaging; ^13^ GRE—Gradient Echo; ^14^ SPGR—Spoiled Gradient Echo; ^15^ FSPGR—Fast Spoiled Gradient Echo.

**Table 2:** Descriptive characteristics of the patients in the post-COVID-19 headache group.

Variable	Count or Mean	±SD
Number of subjects (males)	30 (10)	
Age	55.03 years	15.51 years
Time between COVID infection and MRI	6.1 months	5.1 months
Other post-COVID-19 symptoms, n		
Vertigo	10 (33%)	
Cognitive disorders	9 (30%)	
Visual impairment	3 (10%)	
MR results, n		
White matte lesion	15 (50%)	
Cortical atrophy	7 (23%)	
Vascular encephalopathy	7 (23%)	
Vascular lesion	2 (7%)	
Lacunar lesion	2 (7%)	
Sinusitis	2 (7%)	

**Table 3:** Descriptive characteristics of the control subjects.

Variable	Count or Mean	±SD
Number of subjects (males)	30 (15)	
Age	61.07 years	17.48 years
MR results, n		
Sinusitis	11 (37%)	
Cortical atrophy	8 (27%)	
White matte lesion	6 (20%)	
Vascular encephalopathy	5 (17%)	
Vascular lesion	4 (13%)	
Lacunar lesion	2 (7%)	

**Table 4:** Incidence of observed structural changes.

Results	Post-COVID-19 Headache Group (%)	Control Group (%)
White matter lesion	50	20
Cortical atrophy	23.3	26.7
Vascular encephalopathy	23.3	16.7
Vascular lesion	6.7	13.3
Lacunar lesion	6.7	6.7
Sinusitis	6.7	36.7

**Table 5:** Final diagnosis after MRI.

Final Diagnosis	%
Headache, migraine	23.3
Post-COVID-19 headache	20
Vestibular syndrome	13.3
Anxiety, other psychological disorders	6.7
Sleep disorder, fatigue	6.7
Visual impairment	3.3
Dementia	3.3
Not specified	20
Other	3.3

**Table 6:** Association between MRI findings and diagnoses in the post-COVID-19 headache group.

	White Matter Lesion	Cortical Atrophy	Vascular Encephalopathy	Vascular Lesion	Lacunar Lesion	Sinusitis
**Headache, migraine**	57.1%	28.5%	28.5%	-	-	14.3%
**Post-COVID-19 headache**	50%	33.3%	33.3%	-	16.7%	16.7%
**Vestibular syndrome**	50%	-	25%	-	25%	-
**Anxiety, other psychological disorders**	50%	-	-	-	-	-
**Sleep disorder, fatigue**	50%	-	50%	-	-	-
**Visual impairment**	-	100%	-	-	-	-
**Dementia**	100%	100%	100%	100%	-	-
**Other**	100%	100%	-	100%	-	-

**Table 7:** Treatments.

Treatments	%
Cognitive function stimulant drugs	23.3
Anxiolytic, hypnotic, antidepressant drugs	20
Lifestyle advance	10
Anti-inflammatory drugs	10
Antiepileptic drugs	3.3
Migraine medication	3.3
Vitamin, mineral	3.3
Other drugs	6.7

## Data Availability

Authors are committed to making the data available upon request.

## Abbreviations

The following abbreviations are used in this manuscript:

COVID-19	Coronavirus Disease 2019
CT	Computed Tomography
PET	Positron Emission Tomography
MRI	Magnetic Resonance Imaging
FLAIR	Fluid-Attenuated Inversion Recovery
DWI	Diffusion Weighted Imaging
TOF	Time-of-Flight
SWI	Susceptibility Weighted Imaging
TR	Repetition Time
ms	milliseconds
TE	Echo Time
TI	Inversion Time
FOV	Field of View
mm	millimeters
FS	Fat Saturation
min	minutes
NEX	Number of Excitations
s/mm^2^	seconds per square millimeter
kHz	kilohertz
FSE	Fast Spin Echo
SE EPI	Spin Echo Echo Planar Imaging
GRE	Gradient Echo
SPGR	Spoiled Gradient Echo
FSPGR	Fast Spoiled Gradient Echo
STRIVE	Standards for Reporting Vascular Changes on Neuroimaging
MRA	Magnetic Resonance Angiography
MSKT	Magyar Stroke Konszenzus Tanács (Hungarian Stroke Consensus Panel)
ACR	American College of Radiology
GDPR	General Data Protection Regulation
HUF	Hungarian Forint
